# Abiraterone-Induced Endocrinopathies

**DOI:** 10.1210/jcemcr/luad039

**Published:** 2023-04-13

**Authors:** Scarlett Decamps, Rita Lis, Preethika Ekanayake

**Affiliations:** Division of Endocrinology, Diabetes and Metabolism, University of California, San Diego, 9350 Campus Point Drive, La Jolla, CA 92037, USA; Department of Endocrinology, Diabetes and Metabolism, Veterans’ Affairs, 3350 La Jolla Village Drive, San Diego, CA 92161, USA; Division of Endocrinology, Diabetes and Metabolism, University of California, San Diego, 9350 Campus Point Drive, La Jolla, CA 92037, USA; Department of Endocrinology, Diabetes and Metabolism, Veterans’ Affairs, 3350 La Jolla Village Drive, San Diego, CA 92161, USA; Division of Endocrinology, Diabetes and Metabolism, University of California, San Diego, 9350 Campus Point Drive, La Jolla, CA 92037, USA; Department of Endocrinology, Diabetes and Metabolism, Veterans’ Affairs, 3350 La Jolla Village Drive, San Diego, CA 92161, USA

**Keywords:** abiraterone, CYP17A1 inhibition, mineralocorticoid excess syndrome, secondary adrenal insufficiency, castration-resistant prostate cancer

## Abstract

Abiraterone, a CYP17A1 inhibitor, is used along with prednisone in patients with castration-resistant and castration-sensitive prostate cancer, yielding improved overall and disease-free survival. However, little is documented in the endocrinology literature about the incidences of the endocrine side effects of abiraterone. In this case series, we discuss the diagnosis and management of 3 prostate cancer patients who experienced mineralocorticoid excess and secondary adrenal insufficiency related to abiraterone and prednisone use.

## Introduction

Abiraterone acetate in conjunction with prednisone is increasingly utilized to treat castration-resistant prostate cancer. Although most prostate cancer patients initially respond to androgen deprivation with tumor regression, this effect is often not durable. Progression of prostate cancer is attributed to the reactivation of tumoral androgen receptors (AR) and resurgence of androgen biosynthesis [1]. The multicenter, randomized controlled STAMPEDE trial demonstrated a significantly higher overall and failure-free survival rates in men with locally advanced or metastatic prostate cancer, when abiraterone 1000 mg and prednisone 5 mg were added daily to androgen deprivation therapy (ADT) [2]. The subsequent LATITUDE trial evaluated the same regimen added to ADT in castration-sensitive patients with high-volume metastatic disease [3]. Overall survival benefit was 53.3 months with abiraterone and prednisone compared to 36.5 months with placebo. Furthermore, the risk of progression to castration resistance was lower in the abiraterone/prednisone group, suggesting a reduction of in vitro proliferation and mutation of androgen receptors [4].

**Figure 1.**
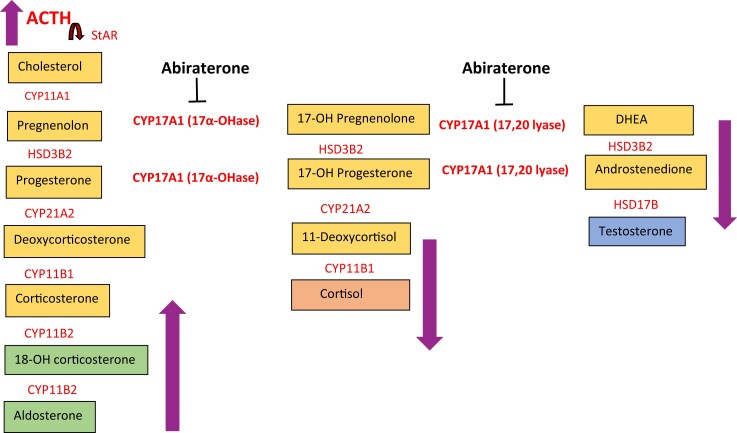
Simplified diagram of steroid synthesis pathway within the adrenal cortex. Abiraterone inhibits the CYP17A1 enzyme, which has 2 different enzymatic activities as 17-alpha hydroxylase and 17,20 lyase. This inhibition leads to relative cortisol deficiency, impairing negative feedback on ACTH. Moreover, it also inhibits androgen production. Excess ACTH continues to drive steroidogenesis with accumulation of steroid hormones ahead of the CYP17A1 inhibition, leading to excess mineralocorticoid formation. The impact of abiraterone's CYP17A1 inhibition on the quantities of mineralocorticoids, cortisol, and androgens is shown by the arrows. Abbreviations: 17α-OHase, 17α-hydroxylase; 18-OH corticosterone, 18-hydroxycorticosterone; CYP, cytochrome p450 enzyme; DHEA, dehydroepiandrosterone; StAR, steroidogenic acute regulatory protein.

Abiraterone is a potent, selective, and irreversible inhibitor of cytochrome P-450c17 (CYP17A1), a critical enzyme in the steroidogenesis pathway. The CYP17 enzyme has 2 distinct roles in steroidogenesis pathway as the 17-alpha hydroxylase and the 17,20 lyase. 17-alpha hydroxylase catalyzes the conversion of pregnenolone to 17-hydroxypregnenolone and the conversion of progesterone to 17-hydroxyprogesterone, which are precursors essential for cortisol production. The inhibition of 17-alpha hydroxylase by abiraterone leads to decreased cortisol production. However, since corticosterone synthesis is unaffected and it also binds and activates the glucocorticoid receptor, manifestations of primary adrenal insufficiency are prevented. The second role of CYP17 enzyme is as 17,20 lyase, which catalyzes the formation of sex steroid precursors (dehydroepiandrosterone and androstenedione) that are ultimately converted to testosterone. Abiraterone's blockage of the 17,20 lyase leads to deficiency in circulating androgens, which benefits patients with prostate cancer (Fig. 1).

CYP17 inhibition by abiraterone leads to loss of negative feedback on the adrenocorticotropic hormone (ACTH), resulting in high levels of ACTH, which cause the formation of excess precursors upstream of the CYP17 inhibition. These precursors are diverted to make excess mineralocorticoids through the uninhibited steroidogenesis enzymes, causing some patients on abiraterone to experience mineralocorticoid excess syndrome (MES). MES, which typically develops by 24 weeks of treatment [5], with clinical findings of hypokalemia, fluid retention, and hypertension, is therefore an on-target side effect of abiraterone. The LATITUDE trial, for example, reported higher incidences of hypertension (41% vs 24%), hypokalemia (24% vs 4%), and fluid retention (14% vs 12%) in the abiraterone/prednisone/ADT group than ADT/placebo group [3].

The addition of a glucocorticoid to abiraterone attempts to attenuate this loss of negative feedback on ACTH to reduce the development and severity of MES. Importantly, lowering excess ACTH also reduces the risk of ACTH-driven androgen formation via a backdoor pathway [1]. When abiraterone was used without a glucocorticoid, 38 out of 42 patients in one study developed MES, with a marked increase in urine mineralocorticoid precursor metabolites. Serum ACTH also had a 660% rise. Although urinary androgen metabolites and serum androgens decreased significantly with single agent abiraterone, there were elevated substrates available for backdoor pathway of dihydrotestosterone (DHT) synthesis [6]. The addition of dexamethasone 0.5 mg to abiraterone reduced urine mineralocorticoid precursors and androgens precursors, including those formed via backdoor pathway. Interestingly, in 9 patients, mineralocorticoid metabolites remained higher than pretreatment baseline. This explains the 15% increase in mineralocorticoid excess seen with abiraterone, even in combination with a glucocorticoid [7].

It is unclear the exact dose of prednisone or dexamethasone that is needed to prevent the occurrence of MES with abiraterone. The large phase 2 and 3 abiraterone trials [2, 3] have mostly used prednisone 5 mg daily. Although dexamethasone is highly effective in reducing MES and back door androgen synthesis, its longer half-life and adverse metabolic effects have caused it to fall out of favor. One randomized control trial demonstrated that a regimen of prednisone 5 mg twice a day added to abiraterone was superior in avoiding MES compared with prednisone 5 mg daily [5]. However, long-term and high doses of exogenous glucocorticoids can cause hypothalamic-pituitary-adrenal (HPA) axis suppression. Some reports have suggested that higher doses of prednisone may be needed during illness in prostate cancer patients taking abiraterone-prednisone to prevent secondary adrenal insufficiency [8]. At this time, real-world incidences of abiraterone-induced MES or secondary adrenal insufficiency are not well described in the endocrinology literature. Here, we present 3 cases of abiraterone-induced endocrine side effects.

## Case Presentations

### Case 1

An 82-year-old patient with prostate cancer with bony metastases on leuprolide, abiraterone 1000 mg, and prednisone 5 mg daily presented to our hospital with lower extremity edema, hypertension, and severe weakness. He recently started furosemide 20 mg for peripheral edema and hypertension.

#### Diagnostic assessment

His systolic blood pressures were between 150 to 179 mmHg and diastolic blood pressures were 73 to 80 mmHg. Labs showed serum potassium of 2.2 mmol/L (2.2 meq/L) with reference 3.5 to 5.1 mmol/L (3.5-5.1 meq/L), random cortisol of 104 nmol/L (3.8 μg/dL) with reference 165.5 to 507.6 nmol/L (6-18 μg/dL), ACTH of 18.26 pmol/L (83 pg/mL) with reference 1.1 to 13.2 pmol/L (5-60 pg/mL), and glomerular filtration rate (GFR) of 31 mL/min/1.73 m [2] (ref >60). Despite stopping furosemide, hypokalemia persisted, and hypertension remained uncontrolled. Aldosterone level was undetectably low and renin activity was suppressed at 0.17 ng/mL/hour (reference 0.25-5.82 ng/mL/hour).

#### Treatment

Due to the high suspicion for abiraterone-induced MES, likely secondary to excess mineralocorticoid precursors activating the aldosterone receptor, abiraterone was held. Prednisone was changed to dexamethasone 1 mg nightly, to avoid further mineralocorticoid receptor (MCR) agonism.

To treat MES, we avoided using the steroid aldosterone receptor blockers like spironolactone and eplerenone, and avoided increasing glucocorticoids, as these have shown to activate wild and mutant types of androgen receptors, potentially worsening castration resistance [4]. Instead, we initiated amiloride 5 mg daily, which selectively blocks the renal epithelial sodium channel, thereby reducing mineralocorticoid sensitive sodium-potassium exchange and sparing potassium excretion. Hydrochlorothiazide 12.5 mg was added to amiloride to enhance the antihypertensive effects as amiloride is a weak antihypertensive. In metabolic balance studies and in clinical studies, amiloride's potassium-sparing effect has been shown to negate the potassium wasting properties of hydrochlorothiazide [9].

#### Outcome and follow-up

Within 48 hours of treatment initiation, the patient's potassium and blood pressure normalized. Given the severity of MES he experienced, the patient opted to stop abiraterone treatment for the foreseeable future. His morning cortisol 4 days after discontinuing all steroids was normal at 383 nmol/L (13.9 μg/dL); reference 165.5 to 507.6 nmol/L (6-18 μg/dL).

### Case 2

The second case involves a 76-year-old male with high-risk metastatic prostate cancer with widespread osseous metastases, on treatment with degarlix (anti-androgen), abiraterone 1000 mg and prednisone 5 mg daily. Three months after initiating abiraterone, the patient presented to the hospital with shortness of breath and malaise and was hypotensive with atrial fibrillation with rapid ventricular response. His blood pressure failed to improve despite hydration and heart rate and rhythm control with cardioversion. He continued to receive his usual daily dose of prednisone 5 mg, though abiraterone was held during illness.

#### Diagnostic assessment

Due to ongoing hypotension, prednisone was held, and a morning cortisol was checked. Cortisol was low at 104.8 nmol/L (3.8 μg/dL) with reference range 165.5 to 507.6 nmol/L (6-18 μg/dL). Therefore, a cosyntropin stimulation test was performed. His next morning cortisol was 226.2 nmol/L (8.2 μg/dL) with ACTH 8.1 pmol/L (37 pg/mL). An hour after cosyntropin injection, cortisol level was 253.8 nmol/L (9.2 μg/dL), suggesting an inadequate response to stimulation.

#### Treatment

The patient's prednisone dose was doubled to 10 mg daily for a few days with resolution of his hypotension. On discharge, abiraterone was not resumed. However, he was instructed to continue prednisone 5 mg daily for the new diagnosis of secondary adrenal insufficiency. A few months later, due to cancer progression, the patient passed away in hospice care.

### Case 3

Similarly, an 87-year-old male with atrial fibrillation, heart failure with preserved ejection fraction, and castrate resistant metastatic prostate cancer with disease progression on ADT was started on abiraterone 1000 mg and prednisone 5 mg daily by oncology. A few months later, he presented to our hospital with abdominal pain and failure to thrive.

#### Diagnostic assessment

Initial workup revealed a hemodynamically stable patient with a slightly low sodium 132 mmol/L or 132 meq/L (ref 135-145 mmol/L or meq/L), potassium 3.6 mmol/L or 3.6 meq/L (ref 3.5-5.1 mmol/L or meq/L), calcium 2.8 mmol/L or 11.4 mg/dL (ref 2.1-2.6 mmol/L or 8.4-10.4 mg/dL), and acute kidney injury with GFR of 8, previously 30 to 40 mL/min/1.73 m [2]. After correcting his electrolyte issues and prerenal kidney injury; and after 8 days of holding prednisone and abiraterone, it was noted that the patient had persistent hypoglycemia (chemistry sugars 2.8 mmol/L or 50 mg/dL), malaise, and hypotension unresponsive to fluids. Morning cortisol values were 104.8 nmol/L (3.8 μg/dL) and 115.9 nmol/L (4.2 μg/dL) on separate occasions, with an inappropriate ACTH of 1.1 pmol/L (5 pg/mL). Subsequently, he also had an inadequate response to cosyntropin stimulation test.

#### Treatment

The patient was discharged on a hydrocortisone taper and kept on physiological doses of hydrocortisone to treat his new secondary adrenal insufficiency. This patient also stopped abiraterone/prednisone treatment for his prostate cancer.

## Discussion

Adverse effects of abiraterone, a CYP17A1 inhibitor, are like that of a rare type of congenital adrenal hyperplasia (CAH), 17-alpha hydroxylase/17,20 lyase deficiency. In the current case series, we presented 3 patients at our institution treated with abiraterone and prednisone for metastatic castration-resistant prostate cancer, with each developing either MES or secondary adrenal insufficiency. Currently there are scant endocrinology publications on the management of these complications in this specific population. With respect to abiraterone-induced MES, similar to previously published reports [10], we have found that the use of amiloride with hydrochlorothiazide is highly effective at minimizing hypokalemia and hypertension symptoms of MES without causing androgen receptor activation, which is undesirable for prostate cancer patients. The newly available nonsteroid, selective mineralocorticoid receptor blockers, such as finerenone and esaxerenone, could also be very useful in treating mineralocorticoid excess with abiraterone. However, neither are currently approved for this indication.

Another potential adverse effect of abiraterone involves the long-term use of prednisone in its treatment regimen, which could incite secondary adrenal insufficiency. Primary adrenal insufficiency is not a concern despite abiraterone's inhibition of the 17-alpha hydroxylase enzyme. As seen in CYP17 deficient CAH, the weak glucocorticoid activity of the mineralocorticoid precursors, especially when their synthesis is exaggerated, typically prevents primary adrenal insufficiency even with reduced cortisol biosynthesis. There are, however, case reports demonstrating prolonged HPA axis suppression, requiring higher doses of glucocorticoids in patients using abiraterone and prednisone, especially during illness [5]. In our case series, 2 patients suffered from secondary adrenal insufficiency during an acute illness, even when abiraterone was being held, with both patients requiring higher doses of glucocorticoids. One hypothesis is that once abiraterone, and therefore the excess mineralocorticoid state, is removed, there are less mineralocorticoids available to activate the glucocorticoid receptor. This, coupled with the protracted suppressive effects of prednisone on the HPA axis, may cause a slow recovery of this axis in these patients. Given the complexity of the hormonal milieu in prostate cancer and the increased risk of endocrinopathies caused by abiraterone/prednisone, endocrinologists should be intimately familiar with diagnosing and safely managing these potential side effects.

## Learning Points

- Abiraterone blocks steroidogenesis at CYP17A1 enzyme, blocking 17-alpha hydroxylase and 17,20 lyase activity of the enzyme. This leads to cortisol deficiency (although excess mineralocorticoids can weakly activate the glucocorticoid receptors), androgen deficiency, and ACTH elevation.
- MES is a common and an on-target side effect of abiraterone even with the use of prednisone 5 to 10 mg daily (15% higher risk of MES).
- Conservative treatment of MES in prostate cancer patients includes using amiloride and hydrochlorothiazide rather than spironolactone and eplerenone, as the latter aldosterone blockers can activate the wild and mutant types of androgen receptors, leading to further castration resistance.
- The use of glucocorticoids in conjunction with abiraterone can lead to HPA axis suppression, resulting in secondary adrenal insufficiency in some patients.

## Contributors

S.D. and R.L. wrote the preliminary drafts of the paper. P.E. finalized the manuscript and completed the formatting of the manuscript and the figure.

## Data Availability

Data sharing is not applicable to this article as no datasets were generated or analyzed during the current study.

## Abbreviations

ACTH: adrenocorticotropic hormone
ADT: androgen deprivation therapy
CYP17: cytochrome P-450c17 (17α-hydroxylase/C17,20-lyase)
HPA: hypothalamic-pituitary-adrenal
MES: mineralocorticoid excess syndrome
